# Supplementary material for: CaMKK2 and CHK1 phosphorylate human STN1 in response to replication stress to protect stalled forks from aberrant resection
Source: Nat Commun. 2023 Nov 30;14:7882. doi: 10.1038/s41467-023-43685-2 (PMC10689503; doi:10.1038/s41467-023-43685-2)
Supplement: Supplementary file 1 — Supplementary Information [file 41467_2023_43685_MOESM1_ESM.pdf]

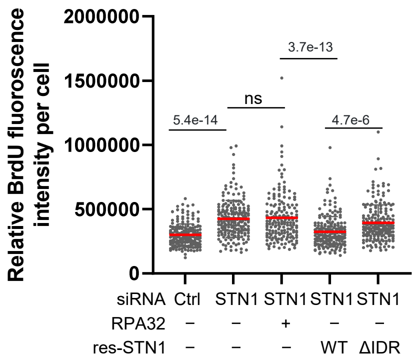

Supplementary Fig. 1. Non-denaturing BrdU analysis. Cells used in Figure 1B were cultured in the presence of 10 µM BrdU for 48 hrs, followed by 4 mM HU treatment for 3 hrs. The cells were then stained with anti-BrdU antibody under non-denaturing conditions to detect ssDNA. Statistical significance was determined by one-way ANOVA. N= Approximately 200 cells were counted for each sample in each experiment. Source data are provided as a Source Data file.

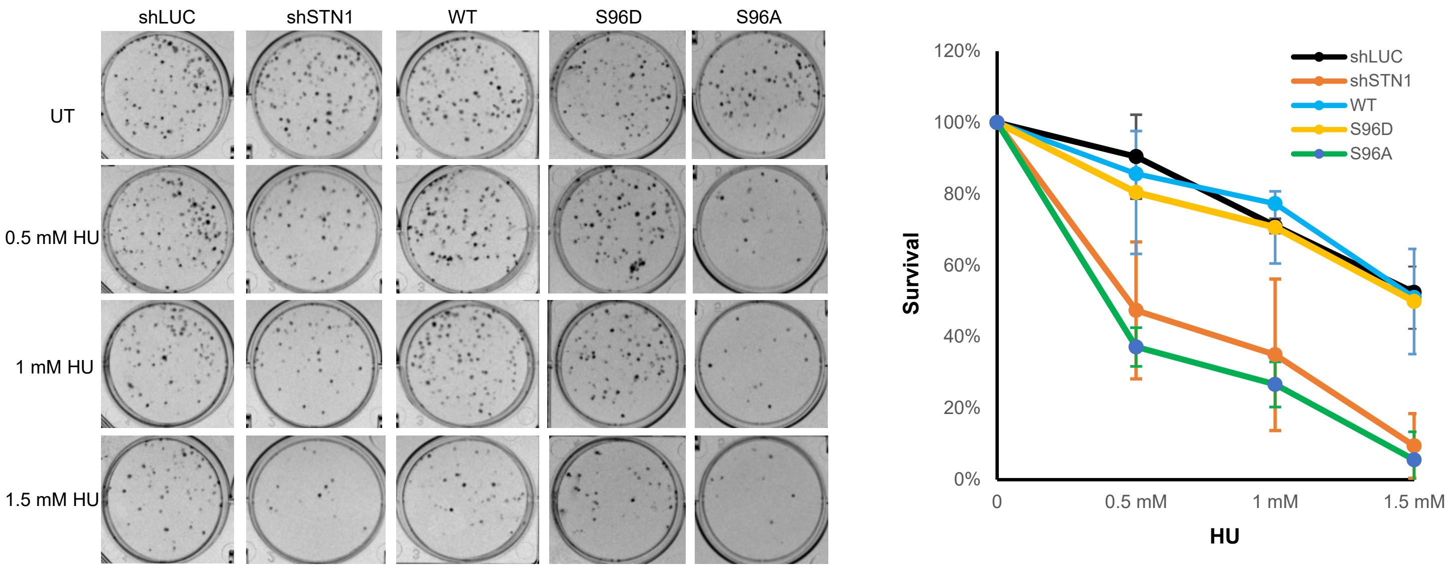

Supplementary Fig. **2. Colony formation assay of HeLa cells expressing WT-STN1, S96D, or S96A with endogenous STN1 depletion**. These cells were treated with 0.5 mM, 1 mM, 1.5 mM of HU for 10 hrs after seeding. Cells were then allowed to grow and form colonies. Colonies were stained and counted. Results are means from two independent experiments. Error bars: SD. Source data are provided as a Source Data file.

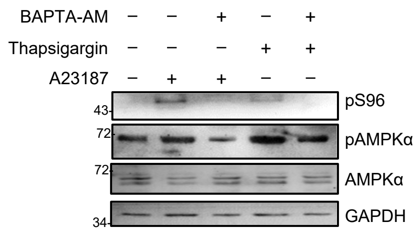

Supplementary Fig. 3. BAPTA-AM treatment impairs S96 phosphorylation after calcium ionophore A23187 and thapsigargin treatment. HeLa cells were pre-treated with BAPTA-AM (50 μM, 30 min) to chelate calcium and then treated with calcium ionophore (A23187, 2 μM, 1 h) or thapsigargin (1 μM, 1 h). STN1 pS96 phosphorylation induced by calcium ionophore and thapsigargin was reduced by BAPTA-AM. Reduction of pAMPKα indicates inhibition of the CaMKK2 signaling. Source data are provided as a Source Data file.

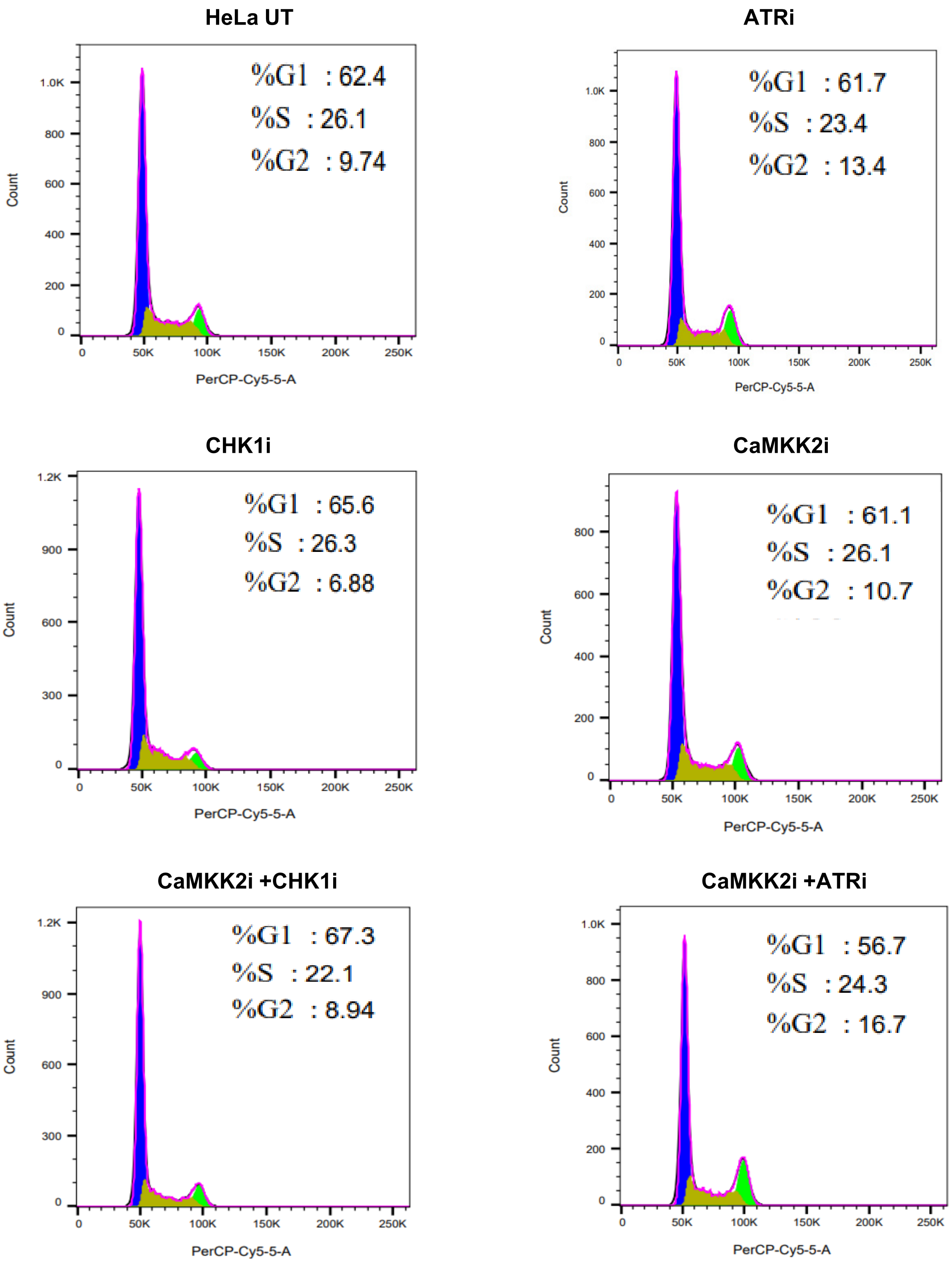

Supplementary Fig. **4. Cell cycle analysis after inhibition of ATR, CHK1, CaMKK2, alone or in combination.** Cell cycle analysis by propidium iodide staining of HeLa cells after inhibition of ATR, CHK1, CaMKK2, alone or in combination using flow cytometry.

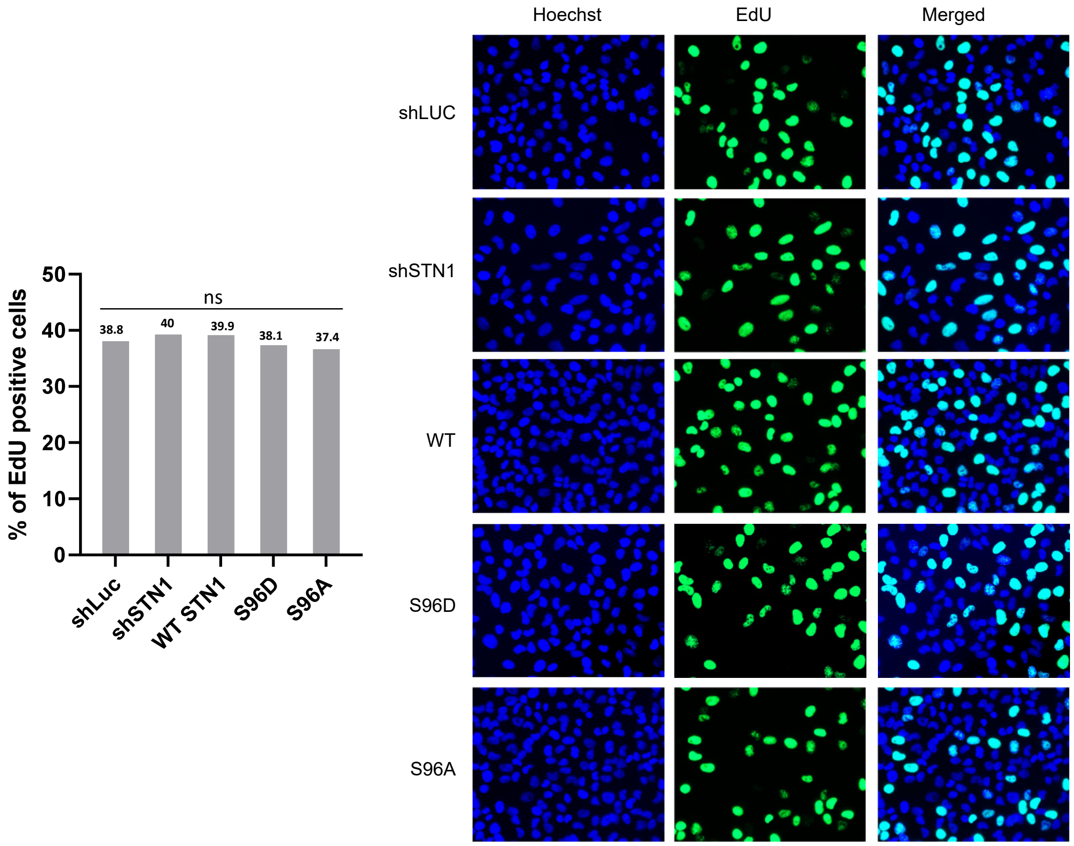

Supplementary Fig. 5. EdU labeling to check the S phase population in cells. EdU labeling in HeLa cells expressing Flag-WT-STN1, S96D, and S96A and with concurrent knockdown of endogenous STN1. Cells were labeled with 10 mM EdU for 2 hr prior to staining. Source data are provided as a Source Data file.

Full blots of Fig. 3A

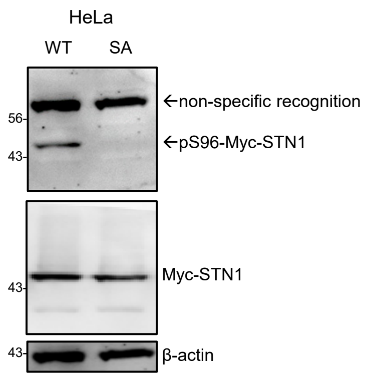

Full blots of Fig. 3B

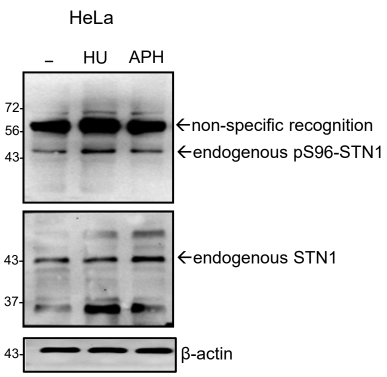

Supplementary Fig. 6. Full blots of Figure 3A and 3B showing that the anti-pS96 antibody exhibits a strong non-specific recognition of a protein bigger than STN1. In the Figure 3A blots, whole cell lysates of HeLa cells transiently expressing Myc-WT-STN1 or Myc-S96A were used on SDS-PAGE and pS96 was detected using the anti-pS96 antibody. Anti-Myc antibody was used to detect Myc-STN1. In the Figure 3B blots, HeLa cells were treated with HU (10 mM, 3 h) and anti-pS96 and anti-STN1 antibodies were used to detect pS96 and endogenous STN1 protein, respectively. Source data are provided as a Source Data file.

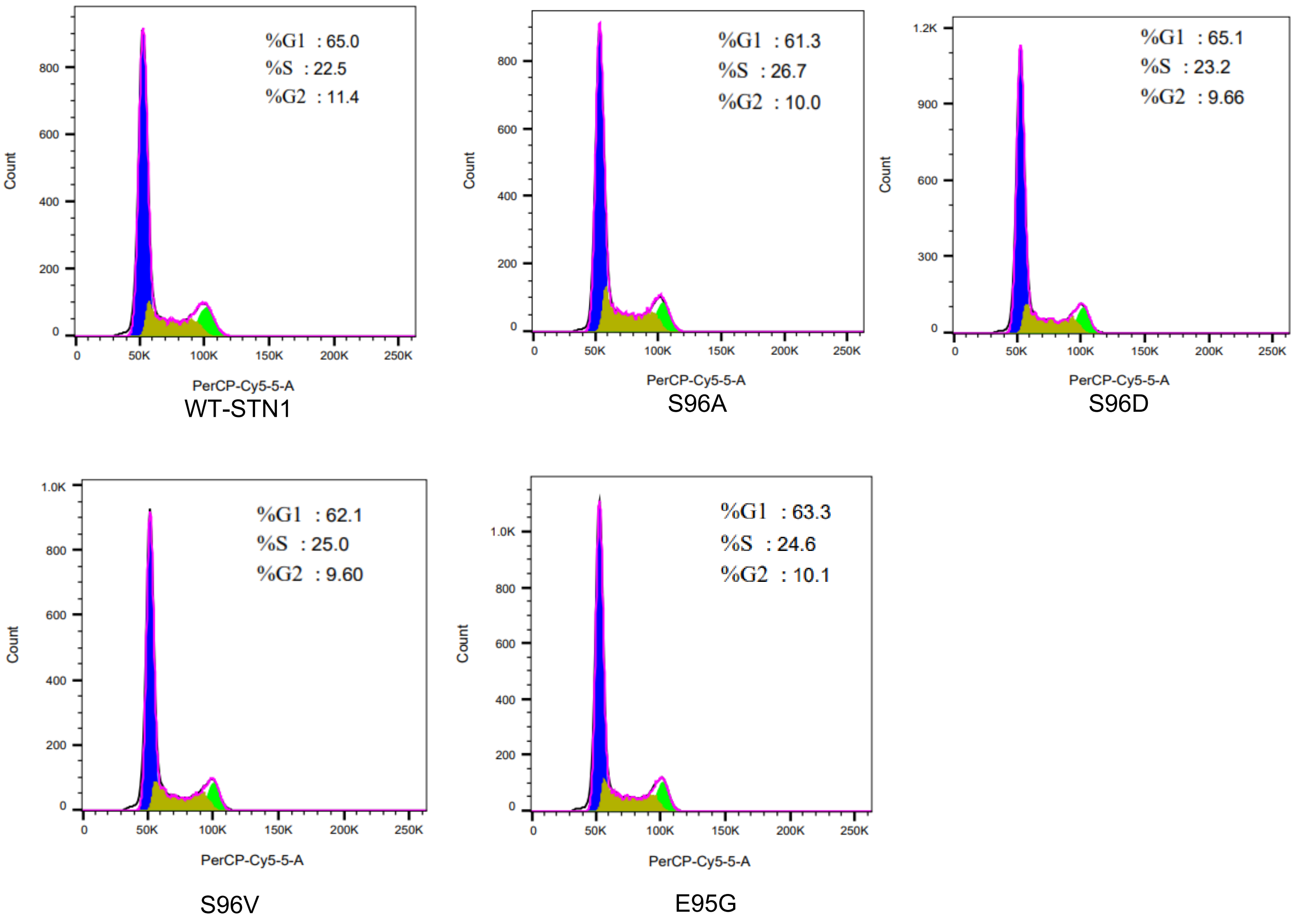

Supplementary Fig. **7. Cell cycle analysis of HeLa expressing RNAi-resistant Myc-WT-STN1, Myc-S96V, Myc-S96A, Myc-E95G with concurrent depletion of endogenous STN1.**

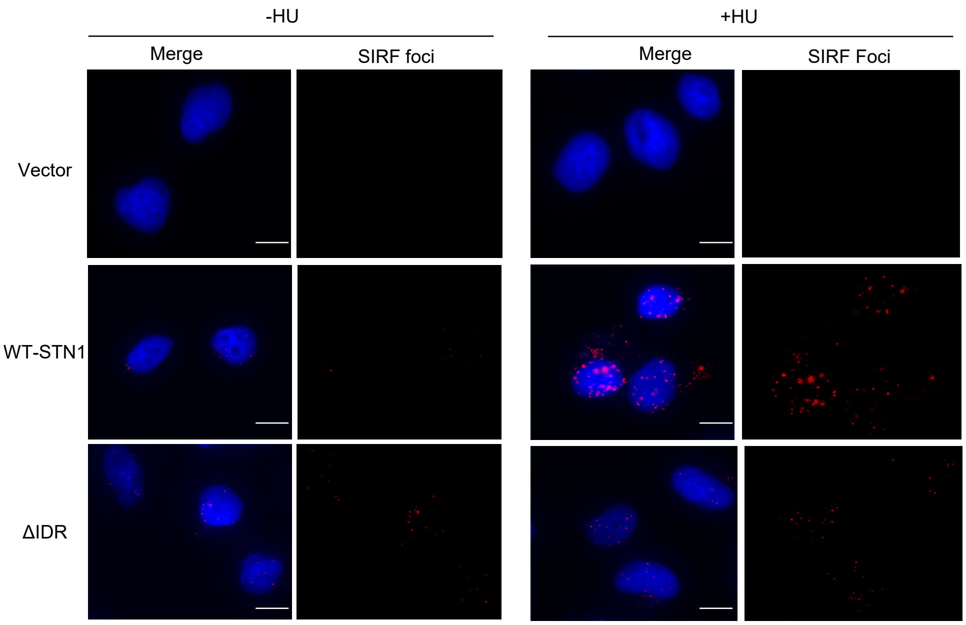

Supplementary Fig. 8 (related to Fig 1E). STN1 SIRF with red channel images.

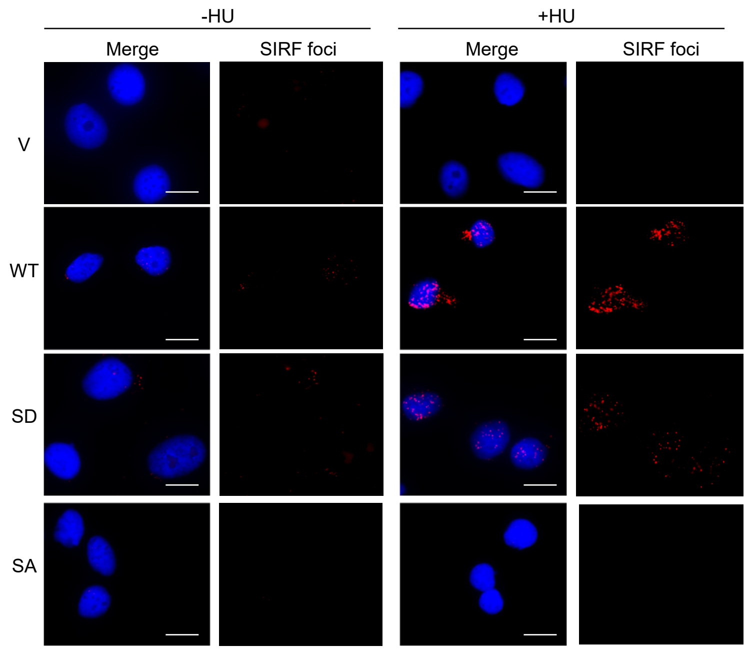

Supplementary Fig. 9 (related to Fig 2B). STN1 mutants SIRF with red channel images.

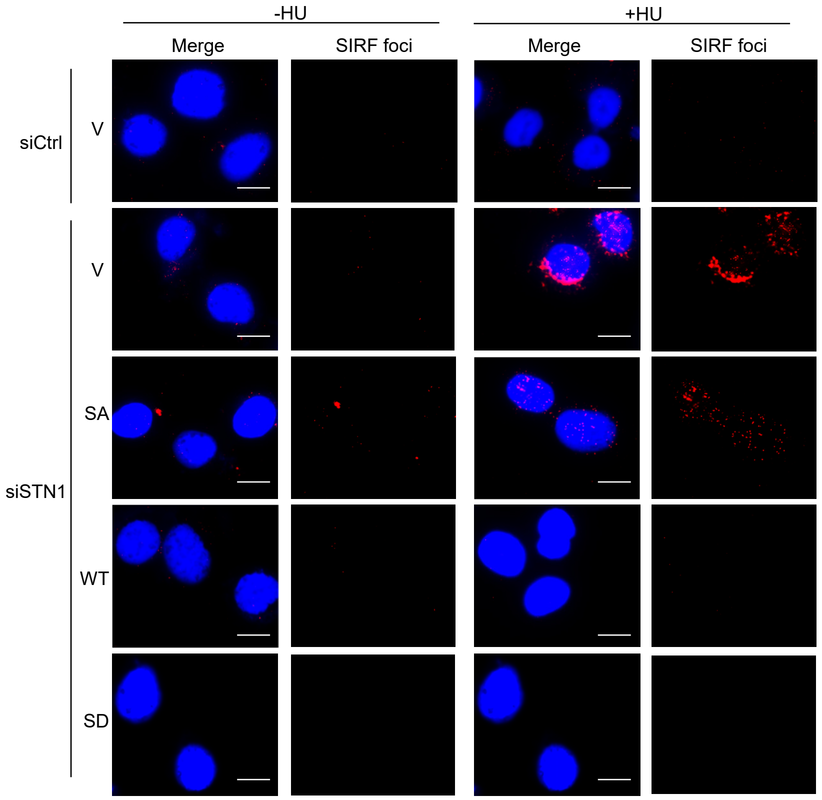

Supplementary Fig. 10 (related to Fig 2E). MRE 11 SIRF with red channel images.

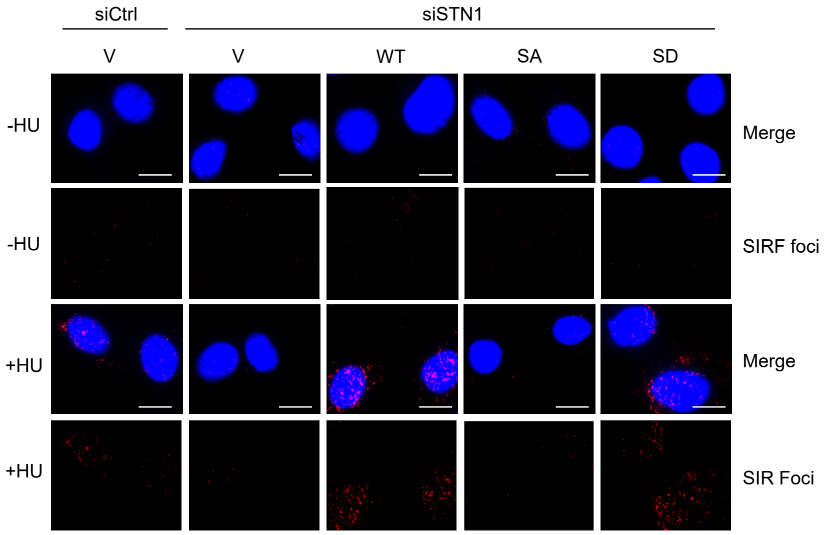

Supplementary Fig. 11 (related to Fig 6A). Rad51 SIRF with red channel images.
