# Supplementary material for: CaMKK2 and CHK1 phosphorylate human STN1 in response to replication stress to protect stalled forks from aberrant resection
Source: Nat Commun. 2023 Nov 30;14:7882. doi: 10.1038/s41467-023-43685-2 (PMC10689503; doi:10.1038/s41467-023-43685-2)
Supplement: Supplementary file 3 — Reporting Summary [file 41467_2023_43685_MOESM3_ESM.pdf]

nature portfolio

Corresponding author(s): Weihang Chai

Last updated by author(s): 2023/11/06

Reporting Summary

Nature Portfolio wishes to improve the reproducibility of the work that we publish. This form provides structure for consistency and transparency in reporting. For further information on Nature Portfolio policies, see our Editorial Policies and the Editorial Policy Checklist.

Statistics

For all statistical analyses, confirm that the following items are present in the figure legend, table legend, main text, or Methods section.

n/a	Confirmed	
☐	☒	The exact sample size (*n*) for each experimental group/condition, given as a discrete number and unit of measurement
☐	☒	A statement on whether measurements were taken from distinct samples or whether the same sample was measured repeatedly
☐	☒	The statistical test(s) used AND whether they are one- or two-sided<br>*Only common tests should be described solely by name; describe more complex techniques in the Methods section.*
☒	☐	A description of all covariates tested
☒	☐	A description of any assumptions or corrections, such as tests of normality and adjustment for multiple comparisons
☐	☒	A full description of the statistical parameters including central tendency (e.g. means) or other basic estimates (e.g. regression coefficient) AND variation (e.g. standard deviation) or associated estimates of uncertainty (e.g. confidence intervals)
☐	☒	For null hypothesis testing, the test statistic (e.g. *F*, *t*, *r*) with confidence intervals, effect sizes, degrees of freedom and *P* value noted<br>*Give P values as exact values whenever suitable.*
☒	☐	For Bayesian analysis, information on the choice of priors and Markov chain Monte Carlo settings
☒	☐	For hierarchical and complex designs, identification of the appropriate level for tests and full reporting of outcomes
☒	☐	Estimates of effect sizes (e.g. Cohen's *d*, Pearson's *r*), indicating how they were calculated

Our web collection on statistics for biologists contains articles on many of the points above.

Software and code

Policy information about availability of computer code

Data collection	ThermoFisher iBright FL1500 Imaging system was used to capture images of all western blots. Blots were developed using SuperSignal West Femto or West Pico Chemiluminescent substrates (ThermoFisher). Images in Figure 7A were captured with Cytiva GE LAS4000 imaging system.<br>Zeiss AxioImager M2 epifluorescence microscope was used to take the SIRF, IF, and DNA fiber images.<br>For measuring the concentration of DNA substrates prepared for in vitro DNA binding and MRE11 degradation assays, the substrate concentration was quantified using absorbance at 260 nm, and the molar extinction coefficients of the substrate with Cy3 were calculated by Molbiotools online software.<br>Live cell imaging of Ca2+ reporters was done using Nikon Ti2E inverted fluorescence microscope.
Data analysis	The ZEN software (Zeiss, Carl Zeiss AG) and GraphPad Prism were used to analyze DNA tract lengths and SIRF.<br>EMSA Gels were analyzed in an Amersham Typhoon 5 Biomolecule imager (Cytiva) with Amersham Typhoon 2.0 software to detect Cy3 fluorescence signal.<br>SDS-PAGE, crystal violet staining, and Coomassie blue staining analyzed the supernatants and eluates to determine protein contents by iBright FL1500 Imaging System (ThermoFisher).<br>MRE11 degradation assay was analyzed by using PRISM 7 software.

For manuscripts utilizing custom algorithms or software that are central to the research but not yet described in published literature, software must be made available to editors and reviewers. We strongly encourage code deposition in a community repository (e.g. GitHub). See the Nature Portfolio guidelines for submitting code & software for further information.

Data

Policy information about availability of data

All manuscripts must include a data availability statement. This statement should provide the following information, where applicable:

- Accession codes, unique identifiers, or web links for publicly available datasets
- A description of any restrictions on data availability
- For clinical datasets or third party data, please ensure that the statement adheres to our policy

Source data for all figures are provided with this paper.

Research involving human participants, their data, or biological material

Policy information about studies with human participants or human data. See also policy information about sex, gender (identity/presentation), and sexual orientation and race, ethnicity and racism.

Reporting on sex and gender	N/A
Reporting on race, ethnicity, or other socially relevant groupings	N/A
Population characteristics	N/A
Recruitment	N/A
Ethics oversight	N/A

Note that full information on the approval of the study protocol must also be provided in the manuscript.

Field-specific reporting

Please select the one below that is the best fit for your research. If you are not sure, read the appropriate sections before making your selection.

☒ Life sciences ☐ Behavioural & social sciences ☐ Ecological, evolutionary & environmental sciences

For a reference copy of the document with all sections, see nature.com/documents/nr-reporting-summary-flat.pdf

Life sciences study design

All studies must disclose on these points even when the disclosure is negative.

Sample size	Three independent experiments were performed for most of the experiments, and at least two independent experiments were performed for the rest. The number of experiments was chosen based on the published literature to generate accurate and reliable results.
Data exclusions	No data were excluded.
Replication	We performed three independent biological repeats in different cell lines to verify the reproducibility of our findings. All attempts at replication were successful.
Randomization	The fluorescence microscope was set to randomly capture images. No randomization was used for western blot and biochemical assays.
Blinding	Blinding is not relevant to our study, as allocating samples into experimental groups was not applicable.

Reporting for specific materials, systems and methods

We require information from authors about some types of materials, experimental systems and methods used in many studies. Here, indicate whether each material, system or method listed is relevant to your study. If you are not sure if a list item applies to your research, read the appropriate section before selecting a response.

Materials & experimental systems

n/a	Involved in the study
☐	☒ Antibodies
☐	☒ Eukaryotic cell lines
☒	☐ Palaeontology and archaeology
☒	☐ Animals and other organisms
☒	☐ Clinical data
☒	☐ Dual use research of concern
☒	☐ Plants

Methods

n/a	Involved in the study
☒	☐ ChIP-seq
☐	☒ Flow cytometry
☒	☐ MRI-based neuroimaging

Antibodies

Antibodies used

The following primary antibodies were used: anti-STN1 (WB 1:1,000, Sigma, WH0079991M1); anti-STN1 (SIRF 1:100, Abcam, ab89250), anti-β-actin (WB 1:60,000, Sigma, A5441), anti-CaMKK2 (WB 1:1000, Santa Cruz, sc-271674), anti-Flag (WB 1:2,000, Sigma, F1804), anti-Myc (WB 1:30,000, Bethyl, A190-105), anti-AMPKα (WB 1:1000, Cell signaling, 2532), anti-HA (WB 1:10,000, Bethyl, A190-108), anti-Chk1 (WB 1:2000, Santa cruz, sc-8408), anti-MRE11 (SIRF 1:200, Abcam, ab214), anti-RPA32 (WB 1:1000, Bethyl, A300-244A), anti-RAD51 (IF 1:10,000. SIRF1:200, Abcam, ab63801), anti-biotin (SIRF 1:100 Millipore-Sigma, SAB4200680), anti-biotin (SIRF 1: 200, Cell Signaling, 5597), anti-GAPDH (WB 1:1000, Cell Signaling, 5174), anti-POLα (WB 1:2000, Bethyl, A302-851), anti-histone H3 (WB 1:3000, Active motif, 61277), anti-pAMPKα-Thr172, (WB 1:1000, Cell Signaling, 2531), anti-AMPKα (WB 1:1000, Cell Signaling, 2532), ATR (WB 1:1000, Cell Signaling, 2790), anti-BrdU (IF 1:200, Roche, 11170376001).

Validation

Validations of all commercial antibodies are based on the datasheets from the manufactures. The primary antibodies were also validated by overexpression or siRNA knockdown of the target gene. All the antibodies used in this study are published in the literature except pS96. We have validated this antibody using western blot.

Eukaryotic cell lines

Policy information about cell lines and Sex and Gender in Research

Cell line source(s)

HeLa, U2OS, 293T,and BJ/hTERT cells were obtained from American Type Culture Collection (ATCC). CaMKK2 KO and AMPKα KO clones were described previously (DOI: 10.1016/j.molcel.2019.04.003)

Authentication

All the cell lines were authorized using Short Tandem Repeats (STRs).

Mycoplasma contamination

All the cell lines were tested negative for Mycoplasma contamination.

Commonly misidentified lines (See ICLAC register)

No commonly misidentified cell lines were used.

Plants

Seed stocks

N/A

Novel plant genotypes

N/A

Authentication

N/A

Flow Cytometry

Plots

Confirm that:

☒ The axis labels state the marker and fluorochrome used (e.g. CD4-FITC).

☒ The axis scales are clearly visible. Include numbers along axes only for bottom left plot of group (a 'group' is an analysis of identical markers).

☒ All plots are contour plots with outliers or pseudocolor plots.

☒ A numerical value for number of cells or percentage (with statistics) is provided.

Methodology

Sample preparation	Propidium iodide (PI) staining was carried out to assess the cell cycle profile using the previously published protocols (93). Briefly, cells were harvested and washed three times with 1x cold PBS, followed by fixation in 70% ethanol for 30 min at 4°C. Propidium stain solution containing 40 µg/ml of PI and 20 µg/ml RNase A in PBS was then added to the fixed cells. Cells were incubated for 15 min at room temperature in the dark. Stained cells were then analyzed using flow cytometer (BD FACS CANTOTM II), with a minimum of 50,000 cells counted for each sample.
Instrument	BD FACS CANTOTM II
Software	FlowJo software was used to analyze the cell cycle distribution.
Cell population abundance	50,000 cells counted for each sample
Gating strategy	We gated for single cells by creating a scatter plot of forward scatter (FSC) vs. side scatter (SSC). Exclude cell aggregates, debris, or other non-single-cell events. We then Created a histogram of PI (or DAPI) fluorescence intensity on a linear scale. This histogram will represent the DNA content of the cells.

☐ Tick this box to confirm that a figure exemplifying the gating strategy is provided in the Supplementary Information.
