# Supplementary material for: CaMKK2 and CHK1 phosphorylate human STN1 in response to replication stress to protect stalled forks from aberrant resection
Source: Nat Commun. 2023 Nov 30;14:7882. doi: 10.1038/s41467-023-43685-2 (PMC10689503; doi:10.1038/s41467-023-43685-2)

Fig 1B

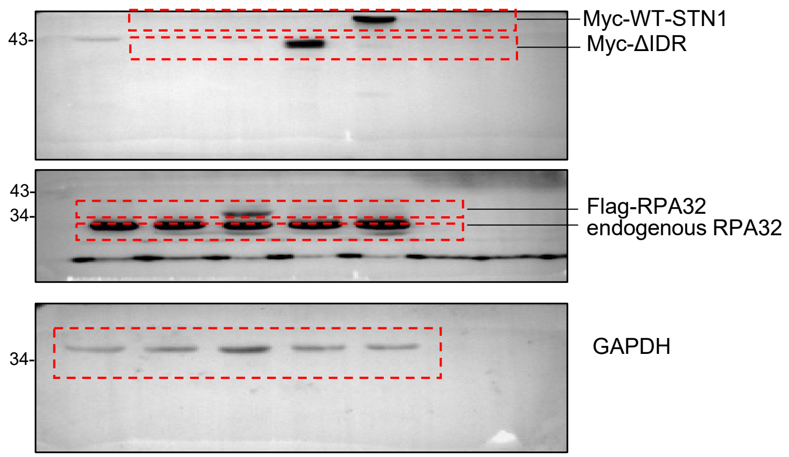
43-
Myc-WT-STN1
Myc-ΔIDR
43-
34-
Flag-RPA32
endogenous RPA32
34-
GAPDH

Fig 2B

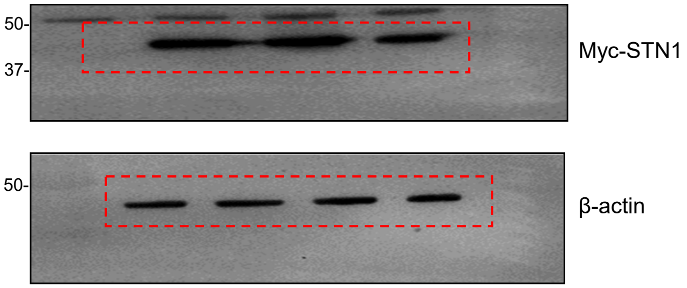

Fig 2C

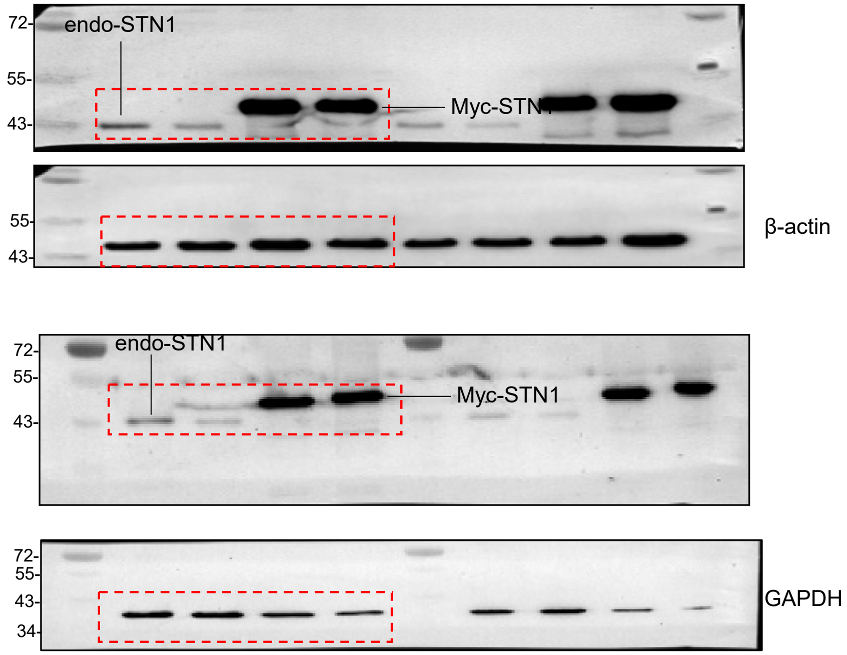

Fig 2D

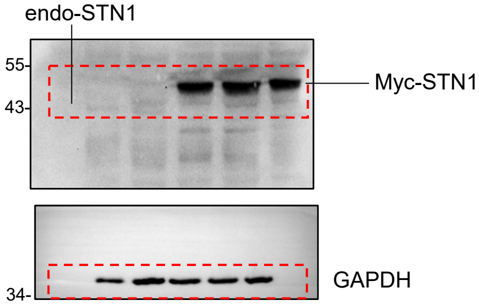

Fig 2E

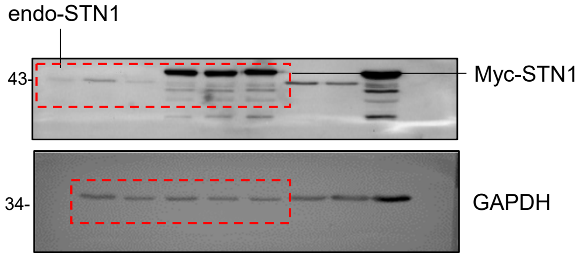

Fig 3A

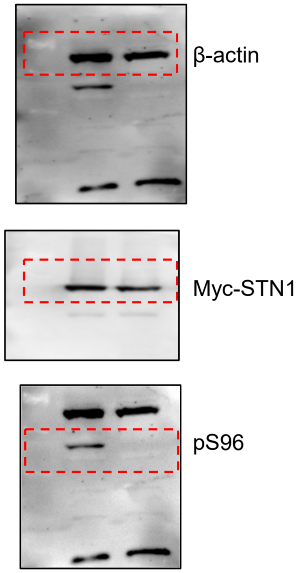
β-actin
Myc-STN1
pS96

Fig 3B

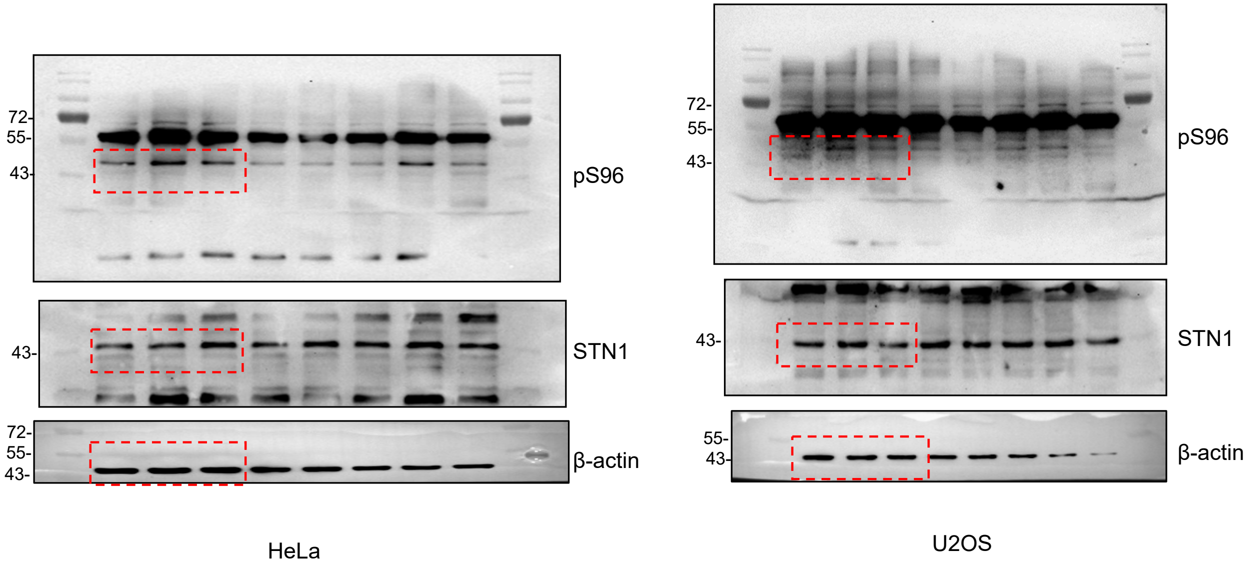
72-
55-
43-
pS96
43-
STN1
72-
55-
43-
β-actin
HeLa
72-
55-
43-
pS96
43-
STN1
55-
43-
β-actin
U2OS

Fig 3C

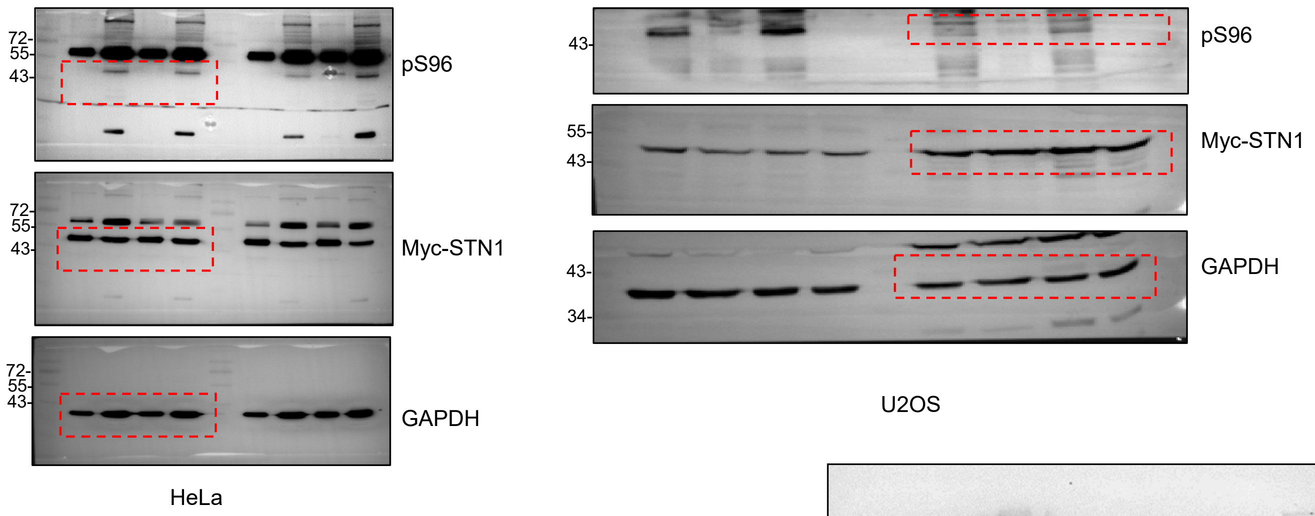
72-
55-
43-
pS96
72-
55-
43-
Myc-STN1
72-
55-
43-
GAPDH
HeLa
43-
pS96
55-
43-
Myc-STN1
43-
34-
GAPDH
U2OS

Fig 3D

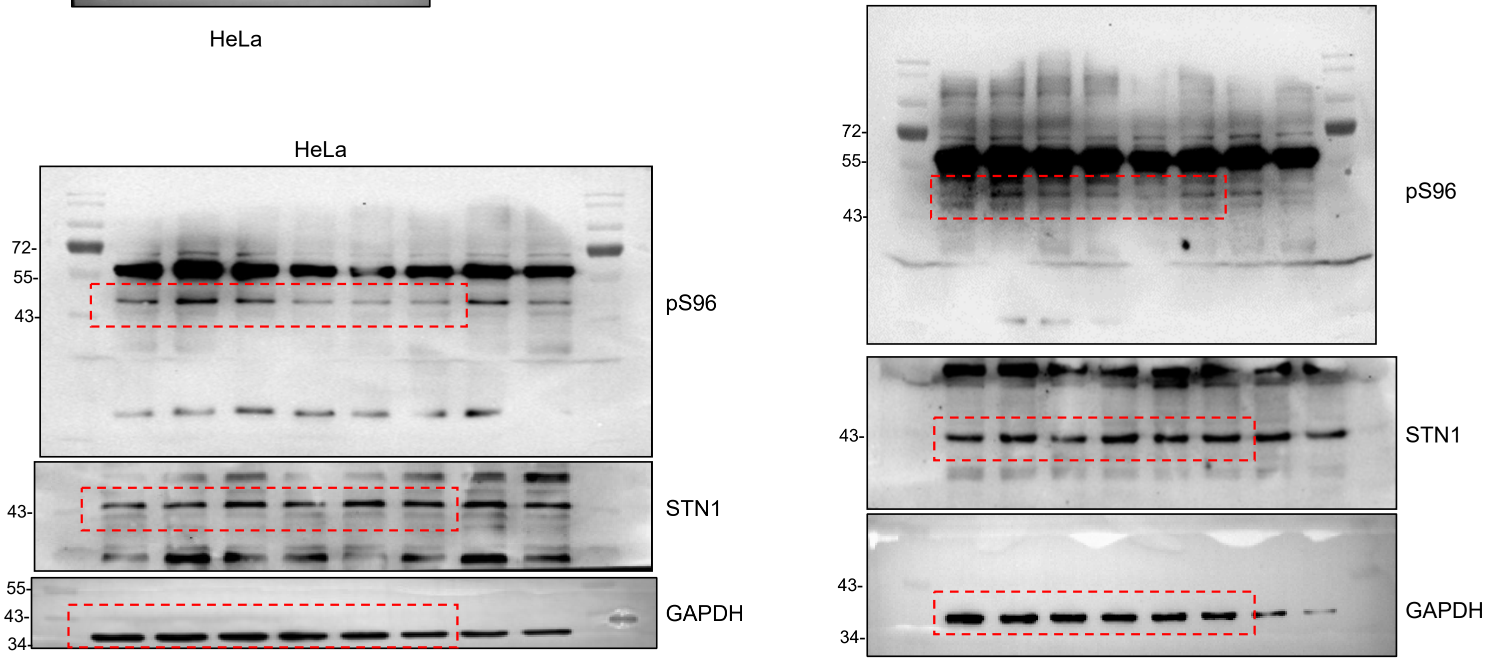
HeLa
72-
55-
43-
pS96
43-
STN1
55-
43-
34-
GAPDH
72-
55-
43-
pS96
43-
STN1
43-
34-
GAPDH

Fig 3E

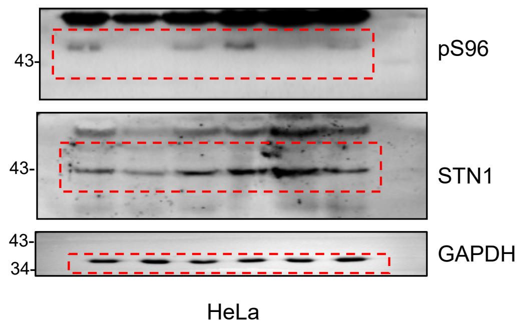

43-
pS96
43-
STN1
43-
34-
GAPDH
HeLa

Fig 3F

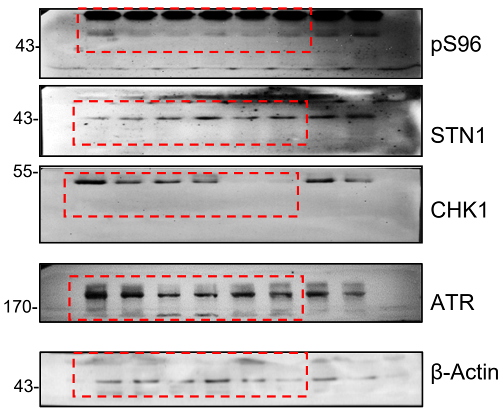

43-
pS96
43-
STN1
55-
CHK1
170-
ATR
43-
β-Actin

Fig 4B

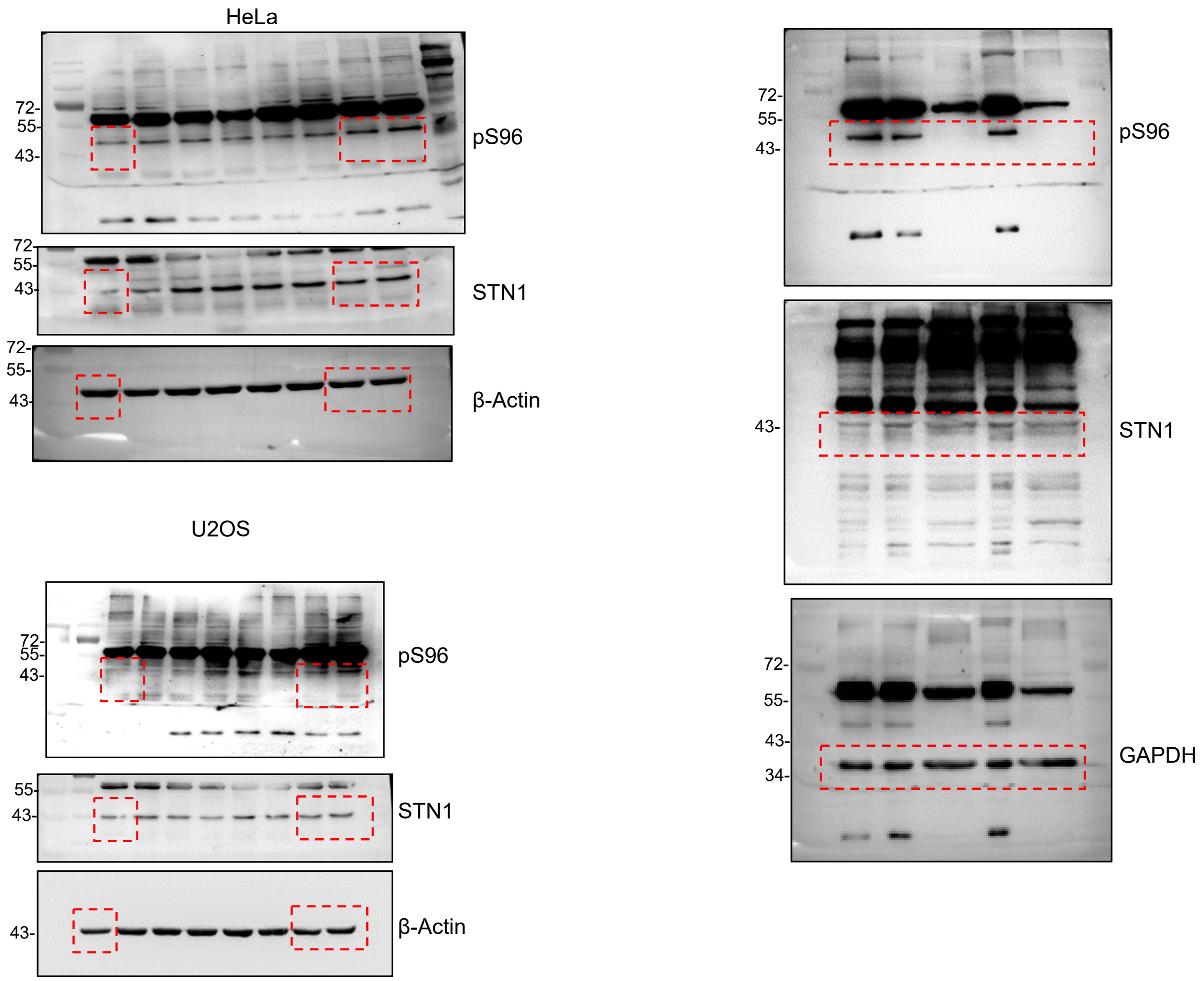
HeLa
72-
55-
43-
pS96
72-
55-
43-
STN1
72-
55-
43-
β-Actin
U2OS
72-
55-
43-
pS96
55-
43-
STN1
43-
β-Actin
72-
55-
43-
pS96
43-
STN1
72-
55-
43-
34-
GAPDH

Fig 4C

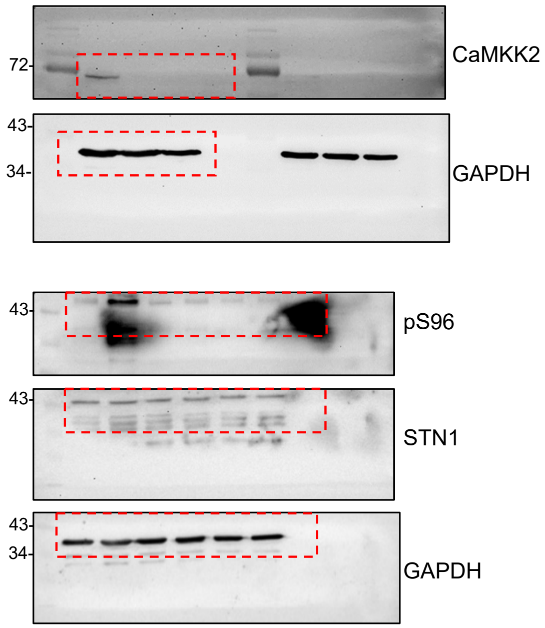

Fig 4D

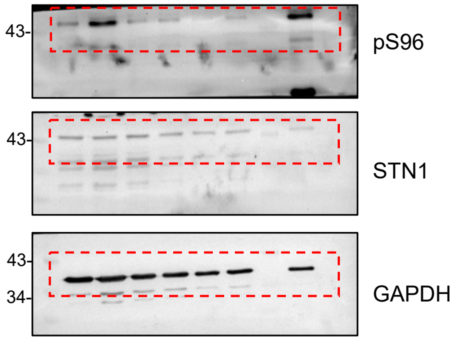

Fig 4E

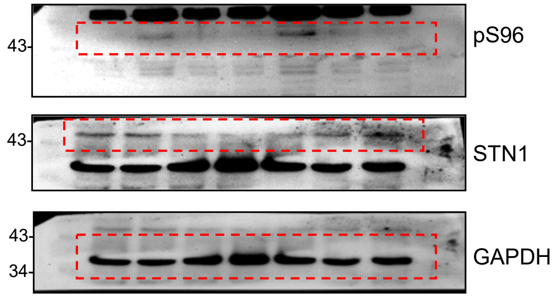

Fig 4F

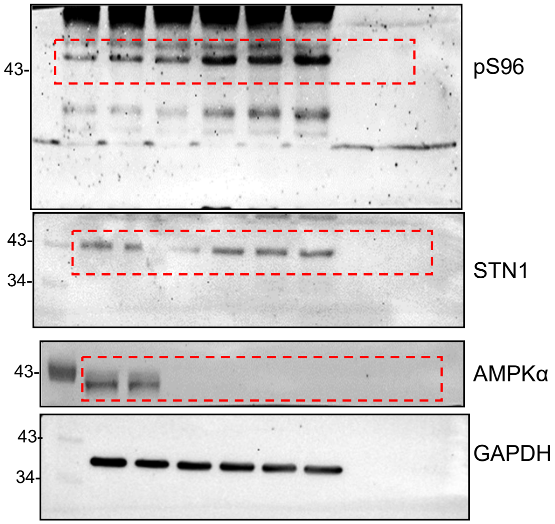

Fig 4G

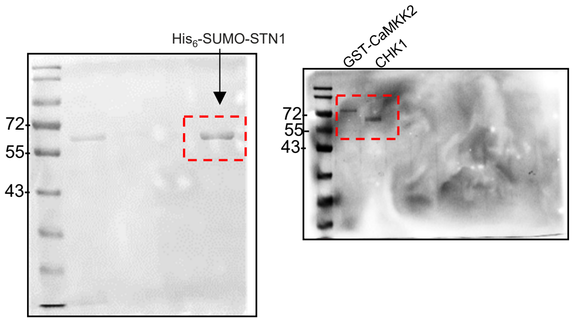

Fig 4H

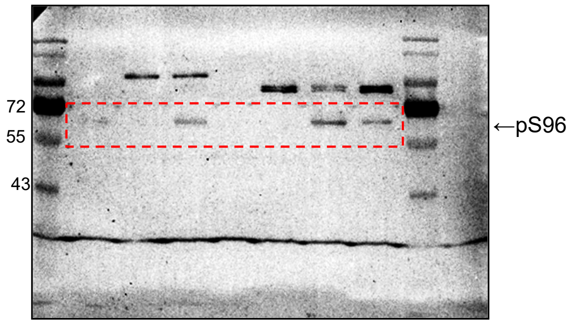

Fig 5A

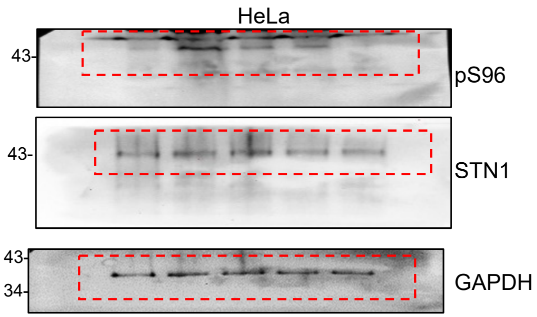
HeLa
43-
pS96
43-
STN1
43-
34-
GAPDH

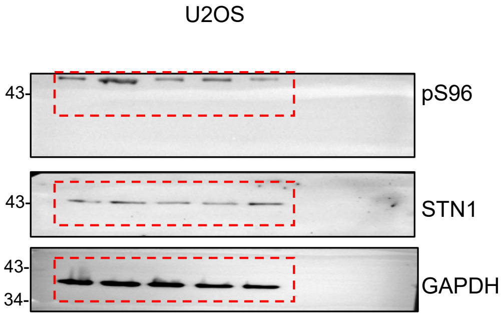
U2OS
43-
pS96
43-
STN1
43-
34-
GAPDH

Fig 6A

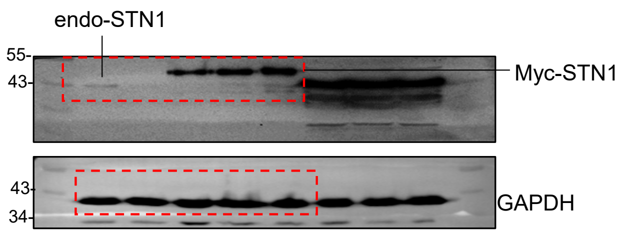

Fig 6C

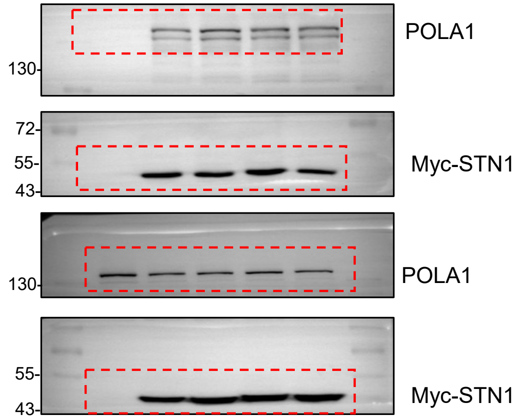

Fig 6D

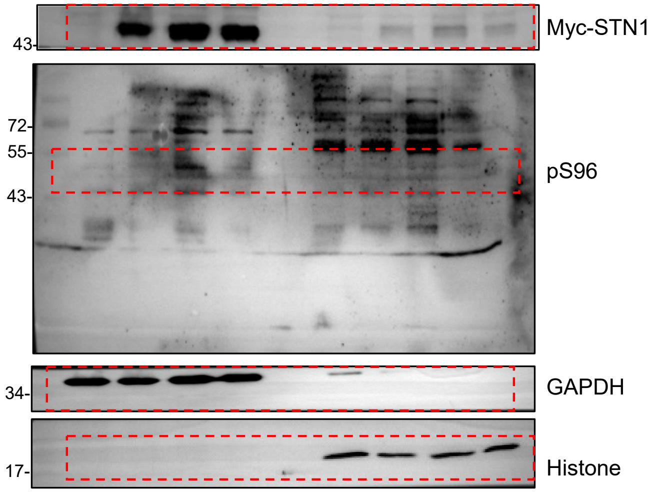

Fig 7A

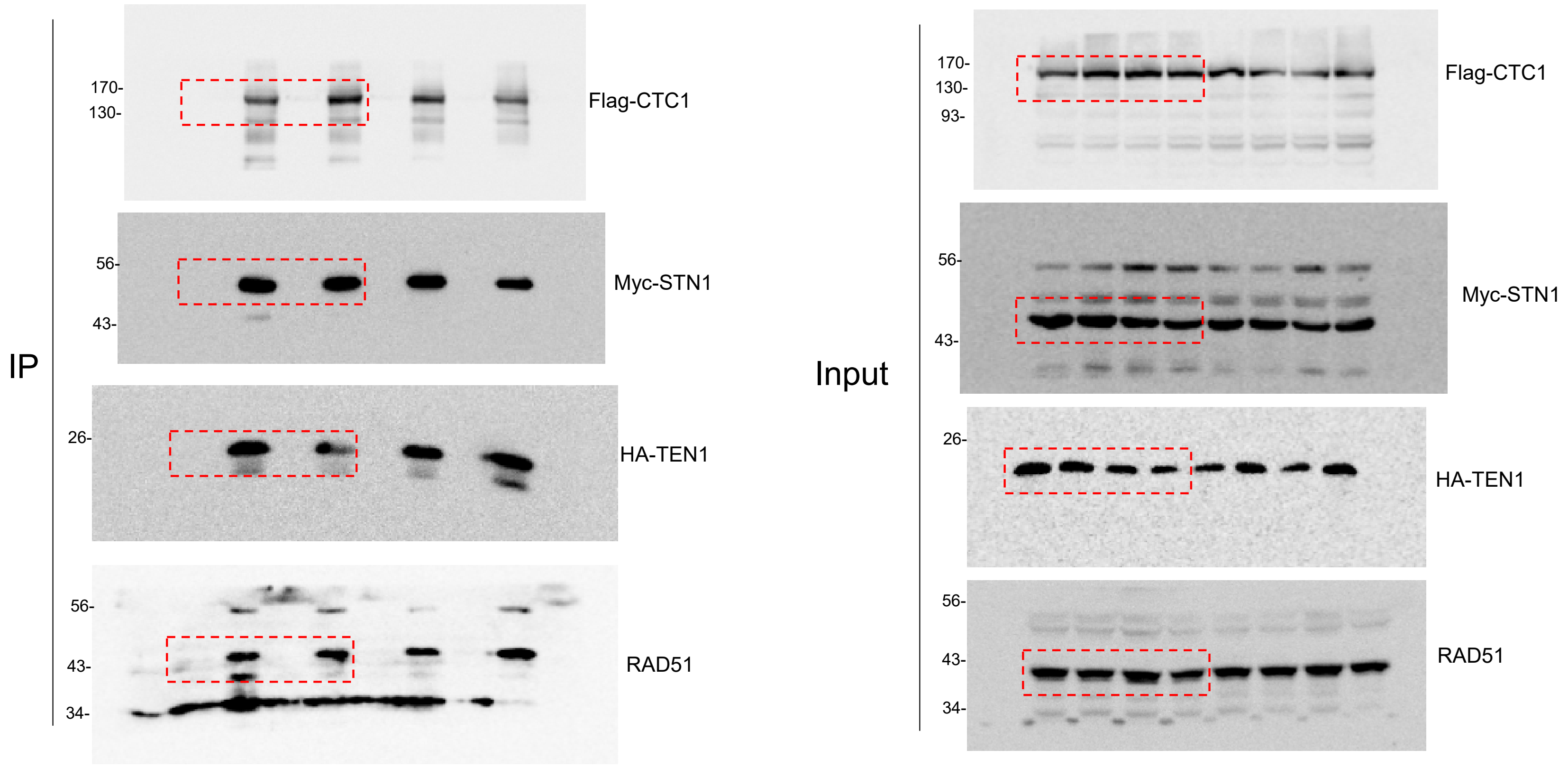
IP
170-
130-
Flag-CTC1
56-
43-
Myc-STN1
26-
HA-TEN1
56-
43-
34-
RAD51
Input
170-
130-
93-
Flag-CTC1
56-
43-
Myc-STN1
26-
HA-TEN1
56-
43-
34-
RAD51

Fig 7B

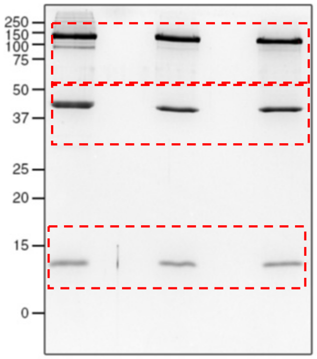

Fig 7C

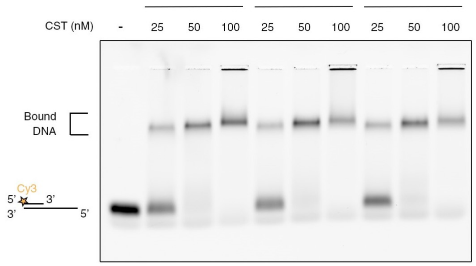

Fig 7D

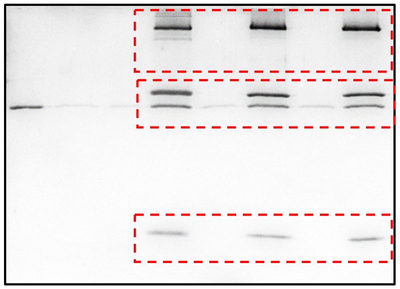

Fig 7E

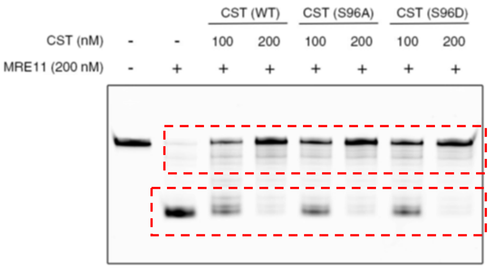

Fig 8A

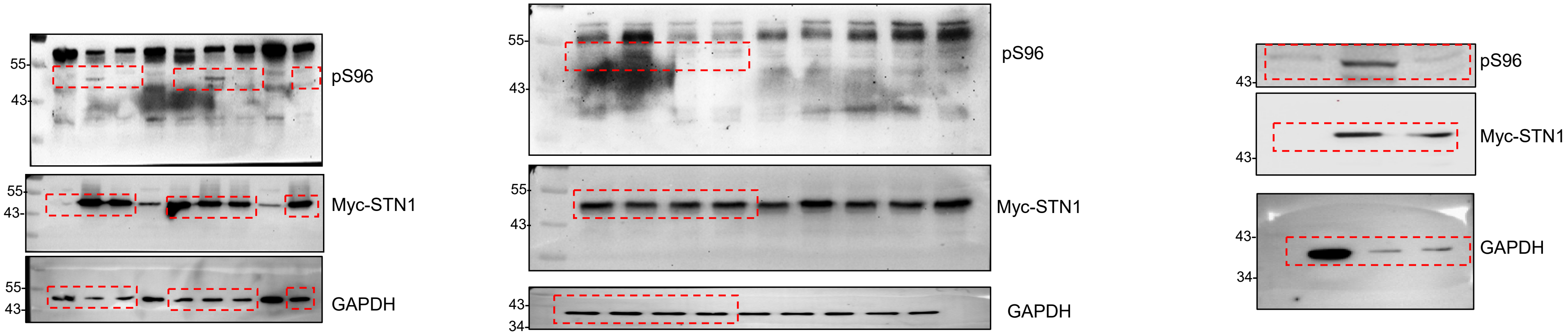

55-
43-
pS96
55-
43-
Myc-STN1
55-
43-
GAPDH
55-
43-
pS96
55-
43-
Myc-STN1
43-
34-
GAPDH
43-
pS96
43-
Myc-STN1
43-
34-
GAPDH

Fig 8B

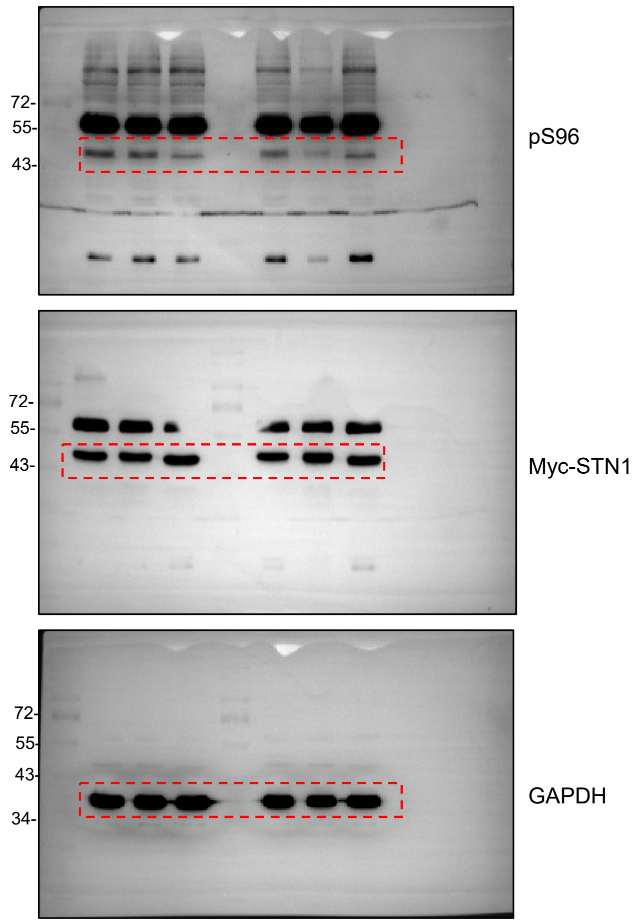

Figure S3.

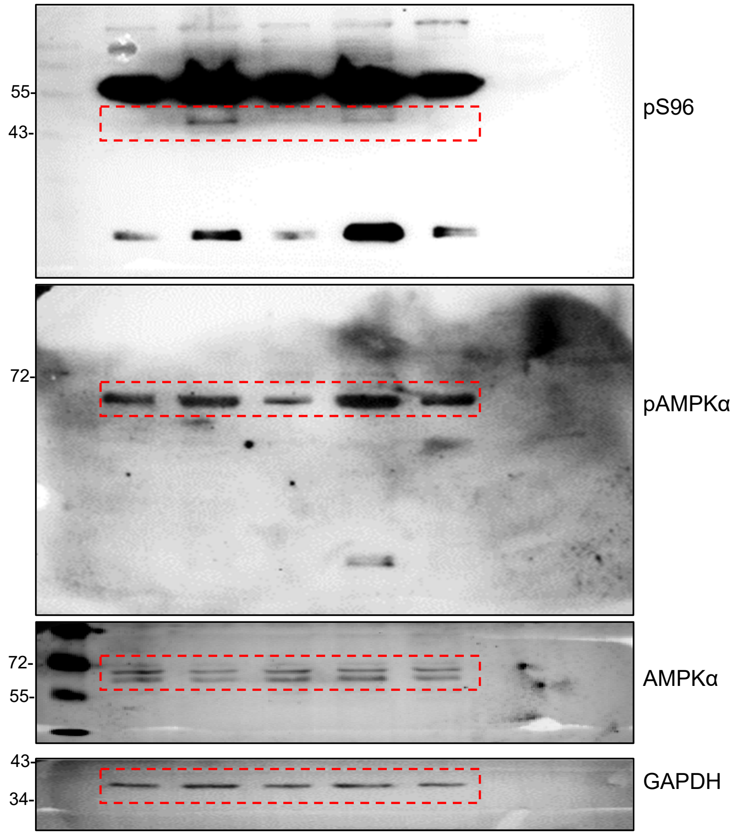

Supplement: Supplementary file 4 — Source Data [file 41467_2023_43685_MOESM4_ESM.zip › Source data file 2_Blots and Gels.pdf]
